# Web-Based Self-Reported Height, Weight, and Body Mass Index Among Swedish Adolescents: A Validation Study

**DOI:** 10.2196/jmir.3947

**Published:** 2015-03-18

**Authors:** Sandra Ekström, Inger Kull, Sara Nilsson, Anna Bergström

**Affiliations:** ^1^Institute of Environmental MedicineKarolinska InstitutetStockholmSweden; ^2^Department of Clinical Science and EducationKarolinska InstitutetStockholmSweden; ^3^Sachs’ Children’s HospitalSouth General Hospital StockholmStockholmSweden; ^4^Center for Occupational and Environmental MedicineStockholm County CouncilStockholmSweden

**Keywords:** adolescent, body height, body weight, body mass index, validity, Internet

## Abstract

**Background:**

Web-collected height and weight are increasingly used in epidemiological studies; however, the validity has rarely been evaluated.

**Objective:**

The aim of the study was to validate self-reported height, weight, and corresponding body mass index (BMI) among Swedish adolescents aged approximately 16 years. A secondary aim was to investigate possible prediction factors for validity of self-reported BMI.

**Methods:**

The study included 1698 adolescents from the population-based cohort BAMSE. Height and weight were collected through a Web-based questionnaire and subsequently measured using standard procedures. Differences between reported and measured height, weight, and corresponding BMI were compared by t tests and agreement was evaluated by Pearson correlation and Bland-Altman plots. Multivariable linear regression analysis was used to investigate whether lifestyle and demographic factors predicted validity of self-reported BMI.

**Results:**

On average, weight was underestimated by 1.1 kg and height was overestimated by 0.5 cm, leading to an underestimation of BMI by 0.5 kg/m2. Correlation coefficients were .98 for height, .97 for weight, and .94 for BMI, and highly significant. Females underestimated weight to a higher extent than males and overweight and obese participants underestimated weight to a higher extent than normal-weight participants, which resulted in higher underestimation of BMI. Underweight participants, on the contrary, overestimated weight and correspondingly BMI. Overall, a high proportion of participants were classified into the correct BMI category; however, among overweight and obese participants, only 60.2% (139/231) and 46% (20/44) were correctly classified, respectively. In the multivariable prediction model, only gender and BMI status significantly predicted discrepancy between reported and measured BMI.

**Conclusions:**

Web-collected BMI may be used as a valid, quick, and cost-effective alternative to measured BMI among Swedish adolescents. The accuracy of self-reported BMI declines with increasing BMI and self-reported BMI should not be used to estimate the prevalence of overweight or obesity.

## Introduction

Self-reported Web-based weight and height have become widely used in epidemiological research as a quick and cost-efficient way to assess body mass index (BMI, kg/m^2^). BMI status is used for investigating disease associations, evaluating interventions, and monitoring obesity trends. The accuracy of Web-based self-reported weight and height is essential; however, few studies have validated these against measured weight and height [1-4].

The validity of self-reported weight and height might vary between populations depending on age, gender, and cultural factors [5-10]. Usually, high correlations between self-reported (using interviews or mailed questionnaires) and measured BMI are observed; however, systematic biases between the two frequently exist. Generally, weight is underestimated whereas height is slightly overestimated, leading to underestimation of BMI and misclassification (eg, of overweight as normal weight) [3,5,6,8]. Overweight/obese people tend to underestimate weight to a higher extent compared to normal-weight people, and women tend to underestimate weight more than men [8,9].

During adolescence, self-reported BMI might be particularly biased. Adolescents experience rapid growth and may have less knowledge about their current weight and height. Also, factors such as body image and social desirability might influence adolescents to report idealized or socially accepted numbers [11].

Using the Web has been suggested to increase the validity of sensitive questions such as weight because it provides anonymity and creates distance between the participant and the researcher [12]. The Web also provides other benefits because it enables direct detection of missing values, elimination of data entry errors, and allows for quick administration to a large number of participants [12]. The Web might be particularly useful for adolescents because they are accustomed to computers and may find Web-based questionnaires easier and more attractive than paper questionnaires. The increasing number of Web-based surveys could, on the other hand, lead to carelessness when filling out these types of questionnaires.

Web-based self-reported height and weight have rarely been evaluated among adolescents [2,13] and, to our knowledge, no study has been done in a European setting. The aim of this study was to validate Web-based self-reported weight, height, and corresponding BMI against measured weight, height, and BMI in Swedish adolescents aged approximately 16 years. A second aim was to investigate determinants for possible discrepancies between self-reported and measured BMI.

## Methods

### Study Design and Study Population

The study population included participants from the prospective birth cohort BAMSE, originally consisting of 4089 children born between 1994 and 1996 in predefined areas of Stockholm. Children in the BAMSE study have been followed repeatedly from birth up to age 16 years, with the main objective to investigate lifestyle and environmental factors associated with the development of allergic diseases. Detailed description of the BAMSE study has been published elsewhere [14,15].

At approximately 16 years of age (range 15.7-19.0 years), adolescents answered a Web-based questionnaire containing questions on various health outcomes and lifestyle factors, including weight and height. After approximately 2 weeks, participants who did not answer the questionnaire were sent a reminder, including a paper-based questionnaire that could be answered instead of the Web-based version. In total, 3115 of 4089 adolescents (76.18%) answered the 16-year follow-up questionnaire of which 2847 (91.40%) answered the Web-based version of the questionnaire.

After answering the questionnaire, participants were invited to a clinical examination where measurements of weight, height, and blood pressure were taken. Weight was measured without shoes and with light indoor clothes to the nearest 0.1 kg using an electronic scale (Seca 799). Height was measured twice without shoes to the nearest 0.1 cm using a wall-mounted wooden stadiometer with a measurement range of 700 to 2050 mm. All measurements were performed by trained nurses and documented according to standard protocols. The study was approved by the regional ethical review board in Stockholm.

### Definition and Classification of Variables

The mean of the 2 height measurements was computed and used in the analyses. BMI was calculated as body weight in kilograms divided by height in meters squared (kg/m^2^). Underweight, normal weight, overweight, and obese were defined using the age- and sex-specific cut-off values developed for children younger than 18 years [16,17]. For participants older than 18 years, underweight was defined as BMI <18.5 kg/m^2^, normal weight as BMI 18.5-24.9 kg/m^2^, overweight as BMI 25-29.9 kg/m^2^, and obese as BMI ≥30 kg/m^2^.

Parental socioeconomic status (professional or manual labor worker) and parental ethnicity (any parent born outside of Scandinavia, including Sweden, Norway, Denmark, or Finland) were obtained at the 8-year follow-up questionnaire in BAMSE. Lifestyle factors and self-rated health were reported by the adolescent in the 16-year questionnaire, whereas blood pressure was measured at the 16-year clinical investigation. Blood pressure was measured 3 times by using an oscillometric monitor (Omron M6 Professional) according to standard procedures recommended by the Swedish Pediatric Society. The mean value of the last 2 measurements was used in the analyses. Questions about physical activity and sedentary time for both summer and winter in the past 12 months were asked. The mean value of the seasons was used in the analyses. Vigorous physical activity included time (hours/week) spent on activities such as lifting heavy weights, aerobics, or high-speed bicycling. Sedentary time included time (hours/day) (outside of school) spent on watching TV, computer use, playing computer or video games, and/or reading. Unreasonable values (>35 hours/week of vigorous physical activity [n=26] and >15 hours/day sedentary time [n=17]) were excluded. Sleep was categorized into an average of <8 or ≥8 hours/night and fruit and vegetable intake were combined into 1 variable that was categorized into every day (≥1/day for fruit and/or ≥7/week for vegetables) or less than every day. Tobacco use included cigarettes and snuffs (regular or irregular use). Self-rated health was categorized into completely healthy compared to fairly healthy/not very healthy. Pubertal status was defined according to a scoring system developed by Petersen et al [18] and categorized into pre/early, mid, or late/post puberty based on questions on body hair development, linear growth spurt, and pubic hair growth (both males and females); voice change and beard growth (males only); and breast development and menarche (females only).

Adolescents (N=1698) where included in the present analyses if (1) self-reported weight and height was reported through the Web (268 paper-based answers excluded), (2) self-reported and measured height and weight were available (additional 471 excluded), (3) self-reported weight and height were collected prior to measured weight and height (additional 19 excluded), (4) the difference between the 2 height measurements was not greater than 0.5 cm (additional 15 excluded), and (5) the time span between self-reported and measured weight and height did not exceed 8 weeks (additional 644 excluded).

### Statistical Methods

Differences in demographic and lifestyle factors were evaluated using the *t* test (continuous variables) and the chi-square test (categorical variables). Mean values of self-reported and measured weight, height, and BMI were compared by using the *t* test and agreement was evaluated by Pearson correlation coefficients. Test for trend of differences across BMI categories were tested using the Wilcoxon rank sum test (Cuzick’s trend test) [19]. Absolute agreement between self-reported and measured BMI was investigated by plotting the difference between self-reported and measured BMI against measured BMI (Bland-Altman plot) [20]. Because weight and height were measured to 1 decimal place but reported without decimals, a sensitivity analysis was performed using measured values of weight and height rounded to 1 decimal place.

To identify factors that might explain differences between self-reported and measured BMI, a prediction model was built by using linear regression with a backward selection technique using a *P* value <.2 from the log-likelihood ratio test to select the final model [21]. The following potential demographic and lifestyle factors, coded and categorized as previously mentioned, were included in the full model: gender, age, parental ethnicity, parental socioeconomic status, BMI status (normal weight as referent), pubertal status, vigorous physical activity, sedentary time, sleep duration, fruit and vegetable intake, tobacco use, and self-perceived health. To be able to compare potential models, only participants with complete information on all the preceding variables were included in the prediction analysis (n=1337).

All analyses were performed using the statistical software Stata version 13 (StataCorp LP, College Station, TX, USA).

## Results

### Description of Study Population

Characteristics of the included study participants are shown in Table 1. Of the 1698 participants, 889 (52.36%) were females and 809 (47.64%) were males. Age when answering the questionnaire ranged from 15.7 to 18.9 years with a mean of 16.5 years and the majority (1517/1698, 89.34%) between 16 and 17 years. The mean time between reported and measured height and weight was 4.6 weeks.

The majority of the participants at 8 years (1458/1619, 90.06%) had at least one parent working as a professional worker and 267 of 1622 (16.46%) had a parent that was born outside of Scandinavia. According to measured weight and height of 1698 participants, 114 (6.71%) were classified as underweight, 1309 (77.09%) as normal weight, 231 (13.60%) as overweight, and 44 (2.59%) as obese.

Compared to females, males were more likely to be overweight and obese and had higher systolic blood pressure. Almost all females (799/813, 98.3%) were in late-/postpuberty, whereas the corresponding proportion for males was 54.8% (382/697). Males reported more vigorous activity, sedentary time, and sleep than females. Less than half of the males (386/799, 48.3%) reported eating fruit or vegetables every day, whereas 64.6% (574/888) of the females did so. Tobacco was used among 238 of 1696 (14.03%) participants and did not differ statistically across gender. The majority of the participants (1382/1693, 81.63%) considered themselves to be completely healthy.

A comparison between the study participants and adolescents that did not fulfill the inclusion criteria (Multimedia Appendix 1) showed that included participants were slightly younger and reported a somewhat lower amount of vigorous physical activity. Also, they considered themselves as completely healthy to a higher extent.

**Table 1:** Description of the study population (N=1698) derived from a prospective birth cohort born in Stockholm in 1994-1996.

Characteristics		All (N=1698)^a^	Females (n=889)^a^	Males (n=809)^a^	*P* ^b^
Age (years),^c^ mean (SD)		16.5 (0.3)	16.5 (0.4)	16.5 (0.3)	.88
Time between self-reported and measured BMI (weeks), mean (SD)		4.6 (1.7)	4.6 (1.7)	4.7 (1.7)	.73
Parental socioeconomic status at 8 years, n (%)					
Professional worker		1458 (90.06)	767 (90.2)	691 (89.9)	.80
Any parent born outside of Scandinavia, n (%)		267 (16.46)	148 (17.4)	119 (15.5)	.31
**BMI status,^d^n (%)**					
	Underweight	114 (6.71)	58 (6.5)	56 (6.9)	
	Normal weight	1309 (77.09)	712 (80.1)	613 (73.8)	
	Overweight	231 (13.60)	104 (11.7)	134 (15.7)	
	Obese	44 (2.59)	15 (1.7)	31 (3.6)	.004
**Blood pressure (mm Hg), mean (SD)**					
	Systolic	121.3 (11.9)	115.9 (9.7)	127.4 (11.2)	<.001
	Diastolic	67.2 (7.0)	67.0 (6.0)	67.3 (7.2)	<.38
**Pubertal status, n (%)**					
	Pre/early	26 (1.72)	1 (0.1)	25 (3.6)	
	Mid	303 (20.07)	13 (1.6)	290 (41.6)	
	Late/post	1181 (78.21)	799 (98.3)	382 (54.8)	<.001
Vigorous physical activity(h/week), mean (SD)		4.8 (4.2)	4.1 (3.6)	5.7 (4.7)	<.001
Sedentary time (h/day), mean (SD)		4.0 (2.2)	3.6 (1.9)	4.4 (2.3)	<.001
Sleep 8 h/day, n (%)		948 (56.03)	462 (52.0)	486 (60.5)	<.001
Fruit and vegetable consumption every day, n (%)		960 (56.91)	574 (64.6)	386 (48.3)	<.001
Tobacco use, n (%)		238 (14.03)	116 (13.1)	122 (15.1)	.22
Consider themselves completely healthy, n (%)		1382 (81.63)	715 (80.5)	667 (82.9)	.23

^a^Numbers for each variable might not add up to total due to missing information in some variables.

^b^ *P* value for comparing females and males.

^c^When answering the questionnaire.

^d^Based on measured weight and height.

### Differences Between Self-Reported and Measured Weight, Height, and Body Mass Index

The mean self-reported and measured height, weight, and corresponding BMI for the total group and separated by gender is shown in Table 2. There were significant differences between self-reported and measured weight, height, and BMI for both girls and boys (*P*<.001). On average, self-reported height was 0.5 cm higher than measured height and self-reported weight was 1.1 kg lower than measured weight. This corresponded to a mean underestimation of BMI by 0.5 kg/m^2^. Females underestimated weight and BMI to a higher extent than males, whereas males overestimated height to a higher extent than females. Using rounded values of measured weight and height resulted in very similar numbers and did not affect the results in any considerable way.

**Table 2:** Mean self-reported and measured height, weight, and BMI by gender.

Anthropometrics		Self-reported, mean (SD)	Measured, mean (SD)	Difference, mean (SD)	*P*
**Total (N=1698)**					
	Height (cm)	173.6 (9.0)	173.1 (9.0)	0.5 (1.8)	<.001
	Weight (kg)	63.9 (11.0)	65.0 (11.5)	–1.1 (2.9)	<.001
	BMI (kg/m^2^)	21.1 (2.8)	21.6 (3.1)	–0.5 (1.1)	<.001
**Females (n=889)**					
	Height (cm)	167.9 (6.1)	167.4 (6.2)	0.4^a^(1.5)	<.001
	Weight (kg)	59.0 (8.7)	60.5 (9.2)	–1.5^b^(2.5)	<.001
	BMI (kg/m^2^)	20.9 (2.8)	21.6 (3.0)	–0.6^b^(1.0)	<.001
**Males (n=809)**					
	Height (cm)	179.9 (7.2)	179.3 (7.2)	0.6 (2.1)	<.001
	Weight (kg)	69.2 (10.7)	69.9 (11.7)	–0.7 (3.2)	<.001
	BMI (kg/m^2^)	21.3 (2.8)	21.7 (3.2)	–0.4 (1.1)	<.001

^a^Significantly different from males (*P*=.02).

^b^Significantly different from males (*P*<.001).

**Figure 1:**
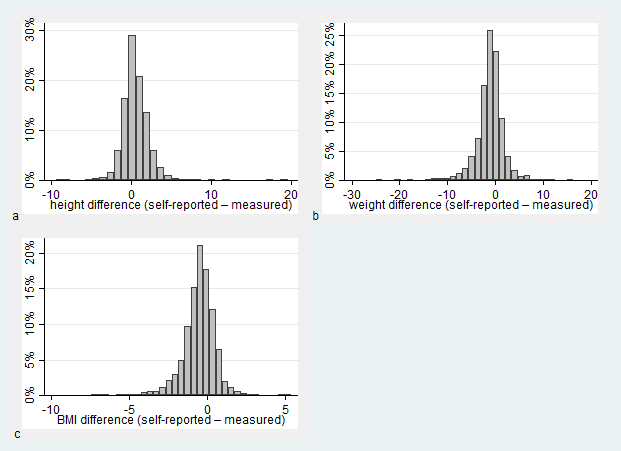
Histogram of difference between self-reported and measured a) height, b) weight, and c) BMI.

Figure 1 shows histograms of differences between self-reported and measured height, weight, and corresponding BMI. Differences ranged from –9.4 to 19.6 cm for height, –24.9 to 16.3 kg for weight, and –7.5 to 5.4 kg/m^2^ for BMI. The differences were relatively normally distributed, although somewhat negatively skewed for weight and BMI. A higher proportion of females compared to males recalled their height within 1 and 2 cm, respectively (550/889, 61.9% and 788/889, 88.6% among females; 415/809, 51.3% and 620/809, 76.6% among males). No such differences were observed for comparable weight or BMI categories (data not shown).

**Figure 2:**
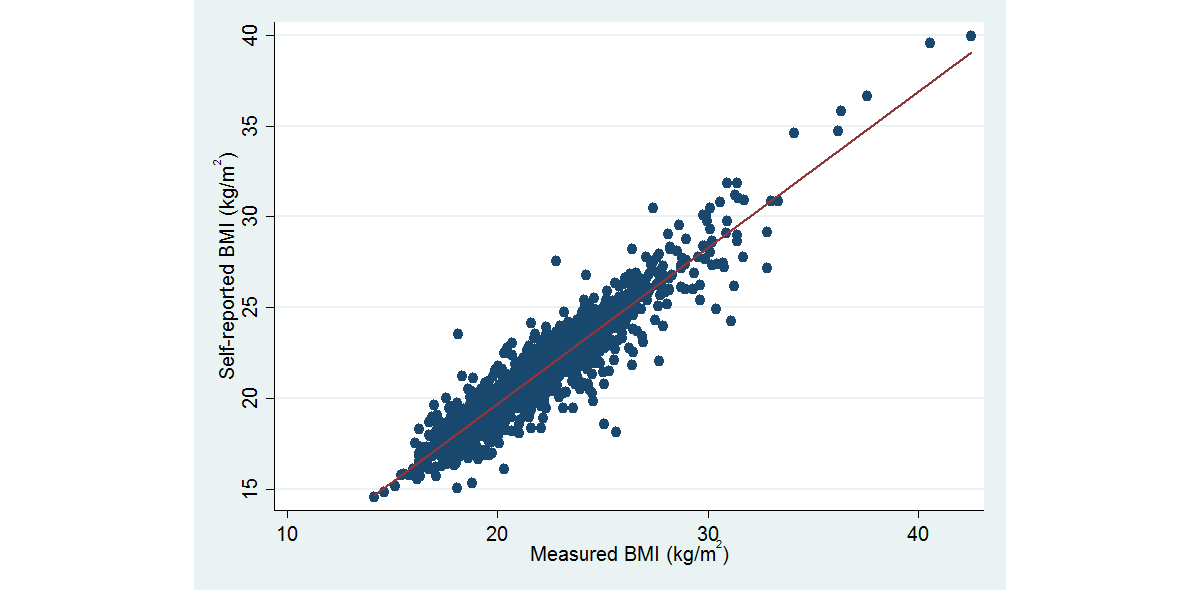
Scatterplot of self-reported (y-axis) and measured (x-axis) BMI (kg/m2), r=.94, P <.001 (N=1698).

The Pearson correlation coefficient between self-reported and measured BMI was .94 (*P*<.001) (Figure 2). For height and weight, the correlation coefficients were .98 and .96, respectively. Similar values were obtained for males and females.

**Figure 3:**
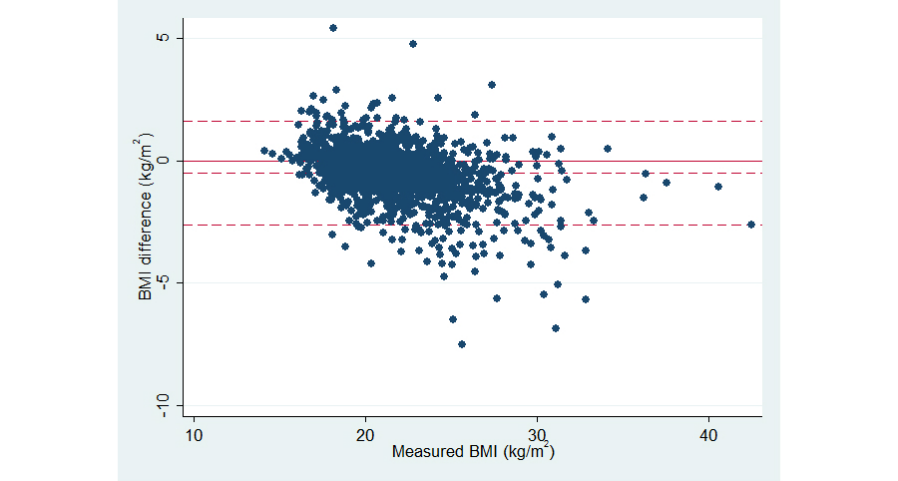
Bland-Altman plot of difference between self-reported and measured BMI (y-axis) in relation to measured BMI (x-axis). The solid line shows difference equal to zero and the dashed lines show mean difference ±2 SD.

Dose-response associations (*P*<.001) between higher BMI categories (defined by measured weight and height) and larger overreporting of height and underreporting of weight and corresponding BMI was observed (Table 3). On average, normal-weight participants underreported weight by 0.8 kg, overweight participants by 2.8 kg, and obese participants by 5.1 kg. In contrast, underweight participants overreported weight by on average 0.9 kg, whereas there was no difference between reported and measured height. Figure 3 shows a graphical illustration of the difference between self-reported and measured BMI in relation to measured BMI (Bland-Altman plot). There was a clear trend of increasing underestimation of BMI with greater measured BMI values.

**Table 3:** Mean of self-reported and measured height, weight, and BMI by measured BMI status.

BMI status^a^		Self-reported, mean (SD)	Measured, mean (SD)	Difference, mean (SD)	*P*
**Underweight (n=114)**					
	Height (cm)	173.1 (8.4)	173.2 (8.7)	0.0 (2.0)	.87
	Weight (kg)	52.1 (6.5)	51.3 (5.4)	0.9 (2. 6)	<.001
	BMI (kg/m^2^)	17.3 (1.0)	17.1 (0.7)	0.3 (0.8)	<.001
**Normal weight (n=1307)**					
	Height (cm)	173.5 (8.9)	173.0 (8.9)	0.5 (1.6)	<.001
	Weight (kg)	61.9 (8.4)	62.8 (8.1)	–0.8 (2.4)	<.001
	BMI (kg/m^2^)	20.5 (1.7)	20.9 (1.7)	–0.4 (0.9)	<.001
**Overweight (n=231)**					
	Height (cm)	174.5 (9.5)	173.5 (9.3)	1.0 (2.0)	<.001
	Weight (kg)	75.5 (9.4)	78.3 (9.0)	–2.8 (3.5)	<.001
	BMI (kg/m^2^)	24.7 (1.8)	25.9 (1.3)	–1.2 (1.3)	<.001
**Obese (n=44)**					
	Height (cm)	175.3 (9.5)	174.2 (9.2)	1.1^b^(2.7)	.009
	Weight (kg)	90.9 (8.9)	96.0 (8.6)	–5.1^b^(5.2)	<.001
	BMI (kg/m^2^)	29.7 (3.5)	31.7 (2.9)	–2.0^b^(1.8)	<.001

^a^Based on measured weight and height.

^b^ *P*<.001 across BMI groups.

Table 4 shows the number of adolescents classified as underweight, normal weight, overweight, and obese according to self-reported and measured BMI, respectively. In total, 1467 of 1698 (86.40%) of the adolescents were classified into the correct BMI category. Among normal-weight participants, 1227 of 1309 (93.74%) were classified correctly, whereas corresponding numbers were 81 of 114 (71.1%) for underweight, 139 of 231 (60.2%) for overweight, and 20 of 44 (46%) for obese. When categorizing participants into nonoverweight or overweight, 1592 of 1698 (93.78%) were classified correctly (1407/1423, 98.88% among nonoverweight and 185/275, 67.3% among overweight).

**Table 4:** Frequencies of children in categories of BMI status according to measured and self-reported weight and height.

Measured	Self-reported, n				Total
	Underweight	Normal weight	Overweight	Obese	
Underweight	81	33	0	0	114
Normal weight	66	1227	16	0	1309
Overweight	0	90	139	2	231
Obese	0	0	24	20	44
Total	147	1350	179	22	1698

### Prediction Factors for Discrepancy Between Self-Reported and Measured Body Mass Index

To identify factors that predict validity of self-reported BMI, a multivariable linear regression model was built using a backward selection method. The final model (n=1337) included gender; BMI categories (based on measured BMI): underweight, overweight, and obese (normal weight as reference); vigorous physical activity (hours/week); and sedentary time (hours/day). Of these variables, only gender and BMI categories significantly predicted differences between self-reported and measured BMI. After mutual adjustment, the estimated mean difference showed that males underreported BMI to a lower extent than females (difference 0.4 kg/m^2^) and that overweight and obese participants underreported BMI to a higher extent compared to normal-weight participants (difference 0.8 and 1.5 kg/m^2^, respectively). Underweight participants, on the contrary, significantly overreported BMI compared to normal-weight participants (difference 0.6 kg/m^2^). Vigorous physical activity and sedentary time were borderline significant predictors of difference between reported and measured BMI (more time associated with larger underreporting), although the absolute effect was very small.

## Discussion

### Principal Findings

We examined the validity of Web-based self-reported weight and height among Swedish adolescents aged approximately 16 years. Correlations between self-reported and measured height and weight were high; however, on average, weight was somewhat underestimated and height was slightly overestimated leading to an underestimation of BMI. Females underreported weight to a higher extent than males and overweight/obese participants underreported weight to a higher extent than normal-weight participants did. Overall, a high proportion of participants were classified into the correct BMI category; however, among overweight and obese participants, only 139 of 231 (60.2%) and 20 of 44 (46%) were correctly classified, respectively. Several lifestyle factors were tested as prediction factors for validity of self-reported BMI; however, after mutual adjustment, only gender and BMI categories significantly predicted discrepancy from measured BMI.

### Comparison With Other Studies

Few studies have evaluated Web-based self-reported weight and height in adolescents. Storey et al [2] validated nutrients, physical activity, weight, and height among 459 adolescents aged 11-15 years in Canada using the Web-based questionnaire Web-SPAN. They found a mean difference between self-reported and measured weight (-2.5 kg), which was larger compared to our study. Similar to us, they found height to be slightly, although nonsignificantly, overestimated. Gender differences and the influence of BMI status were not investigated in this study [2]. Another rather small study [13] validated Web-based weight and height in 137 middle school and 242 high school Korean children. They observed mean differences between self-reported and measured weight and height that were similar to our study (weight difference from -1.1 to -1.7 kg and BMI difference from -0.5 to -0.7 kg/m^2^, depending on gender and school grade). No differences were found between boys and girls. However, the study was not population-based and participants were told beforehand that their weight and height were going to be measured.

The results of our study are also comparable to validation studies using paper questionnaires [10,11,22-26], although varying results have been reported across countries, age groups, and gender. Sherry et al [27] summarized 11 validation studies on US adolescents aged 12-19 years and concluded that self-reported data underestimate overweight prevalence and that there is a gender- and weight status-dependent bias. Compared to our findings, most of the studies included in this review observed somewhat larger discrepancies and lower correlations between estimated and measured weight and height. Two large studies in European settings [11,25], on the other hand, found somewhat smaller differences between reported and measured weight (-0.7 and -0.8 kg) compared to our population.

Most previous studies in adolescents observe larger underreporting of weight and BMI in females compared to males [11,25,28-30]. However, even if validity of self-reported BMI seems to be lower for females, the precision is possibly higher. In this study, this was supported by a lower standard deviation for weight differences and a higher proportion of females that could recall their height within 1 or 2 cm. Fonseca et al [22] similarly found that although mean differences between self-reported and measured BMI were larger in females, variability was lower. Jayawardene et al [26] additionally showed that being female was associated with correct reporting of BMI, whereas being male was associated with overreporting of BMI (using predefined cut-offs for under-, over-, and correct reporting).

Gender differences in the validity of self-reported BMI might have several explanations. Male adolescents experience faster growth and may, therefore, have less knowledge about current weight and height. However, anxiety over their own body size may be more common among females and the social desirability bias might be greater. A more narrow range but larger mean difference between reported and measured BMI would support the hypothesis that females in general are more aware of weight and height than males, although more often systematically underreport weight by 1 or 2 kg.

Several studies [11,13,25,26,31] have observed increasing underreporting with increasing BMI, whereas underweight participants in contrast tend to overreport weight. Consequently, high correlations between self-reported and measured BMI are generally reported whereas the sensitivity for overweight and obesity is low. This indicates that, for this age group, self-reported BMI might be better used as a continuous variable (eg, z score for children younger than 18 years) rather than divided into categories of overweight.

In this study, several lifestyle factors, such as physical activity, fruit and vegetable intake, and tobacco use, were tested as potential explanatory factors for the discrepancy between self-reported and measured BMI. The best fitting model included gender, BMI categories, physical activity, and sedentary time; however, only gender and BMI categories significantly predicted discrepancy between reported and measured BMI. Previous studies have shown conflicting results regarding determinant factors for validity of self-reported BMI. Jayawardene et al [26] found no difference in reporting capacity with physical activity, whereas screen time and fast food consumption were associated with overreporting of BMI. Bae et al [13], on the other hand, found that students who engaged in a high amount of moderate physical activity underestimated weight. Previous studies have shown that self-perceived body image [11,29], weight-loss intentions [13,26], or expressed concern over the own body silhouette and paying close attention to own figure [25] are associated with underreporting. These concerns might, or might not, be independent from overweight and may reflect a tendency to report toward social norms. In this study, we lacked information about these factors; however, we found no association between self-perceived health and reporting capacity.

Differences between studies might be explained by several factors. In this study, there were relatively few overweight participants, which possibly increases validity, compared to populations with higher overweight prevalence. Another factor may be the Swedish tradition of monitoring child growth development through school and health care, which could make families and children more aware of current weight and height. The validity of reported weight and height is likely also affected by the age of the population. Adolescents in the present study were older than in some of the compared studies and perhaps more interested or informed about their weight and height.

### Strengths and Limitations

The main strength of the present study is the population-based design and the relatively large study sample. Height and weight were measured according to standard protocols by trained nurses and were checked for quality controls. In addition, information on a wide range of lifestyle factors (eg, blood pressure, physical activity, and dietary factors) have been collected which made it possible to test whether these affected the validity.

Using the Web for data collection has many advantages, including quick responses, easier administration, and reduction of manual data errors. Web-based questionnaires are more frequently replacing paper-based questionnaires in epidemiological studies; therefore, validation studies are needed to assess the accuracy.

Some limitations need to be addressed. Firstly, measurements were not obtained directly after self-reports. This could have led to small changes in height and weight during the period between the questionnaire and the measurements. To limit bias, we excluded participants with more than 8 weeks between self-reported and measured weight and height.

Secondly, when answering the questionnaire, participants were informed about the clinical investigation. However, it was not stated specifically that the investigation would include height and weight measurements; therefore, it is unlikely to affect the results.

Furthermore, weight was measured with light clothes on. This might explain some of the difference between self-reported and measured weight if participants usually weigh themselves without clothes. Weight could also fluctuate during the day and during the menstrual cycle for females. However, on a group level, these factors would balance each other out.

Lastly, as in all validation studies, the results may not be generalized to other populations as validity might vary with age and ethnicity. To be able to use and interpret self-reported height and weight, it is important to carry out validation studies in different countries and populations. To evaluate whether Web-based questionnaires are more valid than paper questionnaires or interviews, there is a need for more studies, ideally comparing randomized groups of interviews, Web questionnaires, and paper questionnaires.

### Conclusions

Overall agreement between Web-based self-reported and measured weight and height indicate that Web-based BMI can be used as a valid, quick, and cost-effective substitute to measured BMI among Swedish adolescents. The accuracy of self-reported BMI declines with increasing BMI and self-reported BMI should not be used to estimate overweight/obesity prevalences.

## Multimedia Appendix 1

Comparison of lifestyle and demographic characteristics among adolescents included in the study population and total number of adolescents answering the 16 year questionnaire.

## Abbreviations

BMI: body mass index

## References

[1] Pursey K, Burrows TL, Stanwell P, Collins CE (2014). How accurate is web-based self-reported height, weight, and body mass index in young adults?. J Med Internet Res.

[2] Storey KE, McCargar LJ (2012). Reliability and validity of Web-SPAN, a web-based method for assessing weight status, diet and physical activity in youth. J Hum Nutr Diet.

[3] Lassale C, Péneau S, Touvier M, Julia C, Galan P, Hercberg S, Kesse-Guyot E (2013). Validity of web-based self-reported weight and height: results of the Nutrinet-Santé study. J Med Internet Res.

[4] Bonn SE, Trolle Lagerros Y, Bälter K (2013). How valid are Web-based self-reports of weight?. J Med Internet Res.

[5] Huber LRB (2007). Validity of self-reported height and weight in women of reproductive age. Matern Child Health J.

[6] Nyholm M, Gullberg B, Merlo J, Lundqvist-Persson C, Råstam L, Lindblad U (2007). The validity of obesity based on self-reported weight and height: Implications for population studies. Obesity (Silver Spring).

[7] Dijkshoorn H, Ujcic-Voortman JK, Viet L, Verhoeff AP, Uitenbroek DG (2011). Ethnic variation in validity of the estimated obesity prevalence using self-reported weight and height measurements. BMC Public Health.

[8] Wen M, Kowaleski-Jones L (2012). Sex and ethnic differences in validity of self-reported adult height, weight and body mass index. Ethn Dis.

[9] Dahl AK, Hassing LB, Fransson EI, Pedersen NL (2010). Agreement between self-reported and measured height, weight and body mass index in old age--a longitudinal study with 20 years of follow-up. Age Ageing.

[10] Zhou X, Dibley MJ, Cheng Y, Ouyang X, Yan H (2010). Validity of self-reported weight, height and resultant body mass index in Chinese adolescents and factors associated with errors in self-reports. BMC Public Health.

[11] Kurth BM, Ellert U (2010). Estimated and measured BMI and self-perceived body image of adolescents in Germany: part 1 - general implications for correcting prevalence estimations of overweight and obesity. Obes Facts.

[12] van Gelder MM, Bretveld RW, Roeleveld N (2010). Web-based questionnaires: the future in epidemiology?. Am J Epidemiol.

[13] Bae J, Joung H, Kim JY, Kwon KN, Kim Y, Park SW (2010). Validity of self-reported height, weight, and body mass index of the Korea Youth Risk Behavior Web-based Survey questionnaire. J Prev Med Public Health.

[14] Wickman M, Kull I, Pershagen G, Nordvall SL (2002). The BAMSE project: presentation of a prospective longitudinal birth cohort study. Pediatr Allergy Immunol.

[15] Ekström S, Magnusson J, Kull I, Lind T, Almqvist C, Melén E, Bergström A (2015). Maternal body mass index in early pregnancy and offspring asthma, rhinitis and eczema up to 16 years of age. Clin Exp Allergy.

[16] Cole TJ, Bellizzi MC, Flegal KM, Dietz WH (2000). Establishing a standard definition for child overweight and obesity worldwide: international survey. BMJ.

[17] Cole TJ, Flegal KM, Nicholls D, Jackson AA (2007). Body mass index cut offs to define thinness in children and adolescents: international survey. BMJ.

[18] Petersen AC, Crockett L, Richards M, Boxer A (1988). A self-report measure of pubertal status: Reliability, validity, and initial norms. J Youth Adolesc.

[19] Cuzick J (1985). A Wilcoxon-type test for trend. Stat Med.

[20] Bland JM, Altman DG (1986). Statistical methods for assessing agreement between two methods of clinical measurement. Lancet.

[21] Kirkwood BR SJ (2003). Essential Medical Statistics.

[22] Fonseca H, Silva AM, Matos MG, Esteves I, Costa P, Guerra A, Gomes-Pedro J (2010). Validity of BMI based on self-reported weight and height in adolescents. Acta Paediatr.

[23] Rasmussen M, Holstein BE, Melkevik O, Damsgaard MT (2013). Validity of self-reported height and weight among adolescents: the importance of reporting capability. BMC Med Res Methodol.

[24] Legleye S, Beck F, Spilka S, Chau N (2014). Correction of body-mass index using body-shape perception and socioeconomic status in adolescent self-report surveys. PLoS One.

[25] Béghin L, Huybrechts I, Ortega FB, Coopman S, Manios Y, Wijnhoven TMA, Duhamel A, Ciarapica D, Gilbert CC, Kafatos A, Widhalm K, Molnar D, Moreno LA, Gottrand F (2013). Nutritional and pubertal status influences accuracy of self-reported weight and height in adolescents: the HELENA Study. Ann Nutr Metab.

[26] Jayawardene W, Lohrmann D, YoussefAgha A (2014). Discrepant body mass index: behaviors associated with height and weight misreporting among US adolescents from the National Youth Physical Activity and Nutrition Study. Child Obes.

[27] Sherry B, Jefferds ME, Grummer-Strawn LM (2007). Accuracy of adolescent self-report of height and weight in assessing overweight status: a literature review. Arch Pediatr Adolesc Med.

[28] Brener ND, Mcmanus T, Galuska DA, Lowry R, Wechsler H (2003). Reliability and validity of self-reported height and weight among high school students. J Adolesc Health.

[29] Brettschneider AK, Rosario AS, Ellert U (2011). Validity and predictors of BMI derived from self-reported height and weight among 11- to 17-year-old German adolescents from the KiGGS study. BMC Res Notes.

[30] Clarke P, Sastry N, Duffy D, Ailshire J (2014). Accuracy of self-reported versus measured weight over adolescence and young adulthood: findings from the national longitudinal study of adolescent health, 1996-2008. Am J Epidemiol.

[31] Lee B, Chung SJ, Lee SK, Yoon J (2013). Validation of self-reported height and weight in fifth-grade Korean children. Nutr Res Pract.

