# Supplementary material for: Web-Based Self-Reported Height, Weight, and Body Mass Index Among Swedish Adolescents: A Validation Study
Source: J Med Internet Res. 2015 Mar 18;17(3):e73. doi: 10.2196/jmir.3947 (PMC4382564; doi:10.2196/jmir.3947)
Supplement: Supplementary file 1 [file jmir_v17i3e73_app1.pdf]

Multimedia Appendix 1

Validation of web-based self-reported height, weight and BMI among Swedish adolescents

Sandra Ekström, MSc[1]; Inger Kull, PhD[1,2,3]; Sara Nilsson, MSc[4]; and Anna Bergström, PhD[1]

Comparison of lifestyle and demographic characteristics among adolescents included in the study population and total number of adolescents answering the 16 year questionnaire.

	**Included in analyses (n= 1,698)**[a]	**Not fulfilling the inclusion criteria (n= 1,417)**[a]	***P*-value**
**Age** (years)[b], mean (sd)	16.5 (0.3)	16.6 (0.4)	<.001
**Parental socioeconomic status at 8 years**, n (%)			
- Professional worker	1,458 (90.06)	1,174 (89.82)	.84
**Any parent born outside of Scandinavia**, n (%)	267 (16.46)	186 (14.04)	.07
**BMI status**[c], n (%)			
- underweight	114 (6.71)	48 (5.40)	
- normal weight	1,309 (77.09)	683 (76.83)	
- overweight	231 (13.60)	135 (15.19)	
- obese	44 (2.59)	23 (2.59)	.45
**Pubertal status**, n (%)			
- pre/early	26 (1.72)	10 (0.80)	
- mid	303 (20.07)	258 (20.76)	
- late/post	1,181 (78.21)	975 (78.44)	.10
**Vigorous physical** (h/week), mean (sd)	4.8 (4.2)	5.3 (5.0)	0.01
**Sedentary time** (h/day), mean (sd)	4.0 (2.2)	4.0 (2.2)	0.81
**Sleep 8 h/day**, n (%)	948 (56.03)	825 (58.51)	.16
**Fruit and vegetable consumption every day**, n (%)	960 (56.91)	794 (57.16)	.89
**Tobacco use**, n (%)	238 (14.03)	194 (13.77)	.83
**Blood pressure** (mmHg), mean (sd)			
- Systolic	121.3 (11.9)	121.5 (11.4)	0.79
- Diastolic	67.2 (7.0)	67.1 (6.8)	0.84
**Consider themselves completely healthy**, n (%)	1,382 (81.63)	1,107 (78.40)	.003

[a] numbers might not add up to total due to missing
[b] when answering the questionnaire
[c] based on measured weight and height
